# 葡萄糖-6-磷酸脱氢酶缺乏对血液病患者异基因造血干细胞移植的影响

**DOI:** 10.3760/cma.j.cn121090-20231009-00176

**Published:** 2024-02

**Authors:** 佳 王, 海霞 付, 圆圆 张, 晓冬 莫, 婷婷 韩, 军 孔, 于谦 孙, 萌 吕, 伟 韩, 欢 陈, 育红 陈, 峰蓉 王, 晨华 闫, 瑶 陈, 景枝 王, 昱 王, 兰平 许, 晓军 黄, 晓辉 张

**Affiliations:** 1 北京大学人民医院，北京大学血液病研究所，国家血液系统疾病临床医学研究中心，造血干细胞移植治疗血液病北京市重点实验室，北京 100044 Peking University People's Hospital, Peking University Institute of Hematology, National Clinical Research Center for Hematologic Disease, Beijing Key Laboratory of Hematopoietic Stem Cell Transplantation, Beijing 100044, China; 2 安徽医科大学第二附属医院，合肥 230601 The Second Hospital of Anhui Medical University, Hefei 230601, China

**Keywords:** 葡萄糖-6-磷酸脱氢酶缺乏症, 异基因造血干细胞移植, 并发症, 预后, G6PD deficiency, Allogeneic hematopoietic stem cell transplantation, Complication, Prognosis

## Abstract

**目的:**

探讨葡萄糖-6-磷酸脱氢酶（G6PD）缺乏症患者和非G6PD缺乏症患者在异基因造血干细胞移植（allo-HSCT）过程中预处理毒性、移植并发症和生存的差异。

**方法:**

收集2015年3月至2021年1月G6PD缺乏症患者在北京大学人民医院行allo-HSCT的连续病例作为研究组。以1∶5的比例随机抽选同期接受allo-HSCT，且性别、年龄、疾病、移植方式匹配的非G6PD缺乏患者作为对照组。收集两组患者的临床资料，进行回顾性巢式病例对照研究。

**结果:**

共7例G6PD缺乏患者进入研究组，35例非G6PD缺乏患者作为对照组。研究组7例G6PD缺乏患者中男6例，女1例，中位年龄37（2～45）岁，均无明显G6PD缺乏症相关临床症状；血液系统原发病包括急性髓系白血病3例、急性淋巴细胞白血病2例、重型再生障碍性贫血2例。移植后28 d内研究组所有患者均获得粒细胞植入，对照组粒细胞植入率为94.3％；研究组、对照组粒细胞植入中位时间分别为13（11～17）d、12（10～23）d（*P*＝0.601），血小板植入中位时间分别为21（6～64）d、14（7～70）d（*P*＝0.113）。研究组、对照组继发性移植物功能不良（PGF）发生率分别为42.9％（3/7）、8.6％（3/35）（*P*＝0.036），巨细胞病毒（CMV）感染发生率分别为71.4％（5/7）、31.4％（11/35）（*P*＝0.049），出血性膀胱炎发生率分别为57.1％（4/7）、8.6％（3/35）（*P*＝0.005），细菌感染发生率分别为100.0％（7/7）、77.1％（27/35）（*P*＝0.070），EB病毒（EBV）感染发生率分别为14.3％（1/7）、14.3％（5/35）（*P*＝1.000），真菌感染发生率分别为14.3％（1/7）、25.7％（9/35）（*P*＝0.497），移植后淋巴细胞增殖性疾病（PTLD）发生率分别为0％（0/7）、5.7％（2/35）（*P*＝0.387）。

**结论:**

合并G6PD缺乏的血液病患者可耐受常规allo-HSCT预处理方案，粒细胞、血小板可顺利植入，但移植后需警惕病毒感染、出血性膀胱炎及继发性移植物功能不良。

## Abstract (en)

**Objective:**

To determine the effect of glucose-6-phosphate-dehydrogenase (G6PD) deficiency on patients’ complications and prognosis following allogeneic stem cell hematopoietic transplantation (allo-HSCT).

**Methods:**

7 patients with G6PD deficiency (study group) who underwent allo-HSCT at Peking University People's Hospital from March 2015 to January 2021 were selected as the study group, and thirty-five patients who underwent allo-HSCT during the same period but did not have G6PD deficiency were randomly selected as the control group in a 1∶5 ratio. Gender, age, underlying diseases, and donors were balanced between the two groups. Collect clinical data from two patient groups and perform a retrospective nested case-control study.

**Results:**

The study group consisted of six male patients and one female patient, with a median age of 37 （range, 2–45）years old. The underlying hematologic diseases included acute myeloid leukemia（*n*＝3）, acute lymphocytic leukemia （*n*＝2）, and severe aplastic anemia （*n*＝2）. All 7 G6PD deficiency patients achieved engraftment of neutrophils within 28 days of allo-HSCT, while the engraftment rate of neutrophils was 94.5％ in the control group. The median days of platelet engraftment were 21（6–64）d and 14 （7–70）d （*P*＝0.113）. The incidence rates of secondary poor graft function in the study group and control group were 42.9％ （3/7）and 8.6％ （3/35）, respectively （*P*＝0.036）. The CMV infection rates were 71.4％ （5/7）and 31.4％ （11/35）, respectively （*P*＝0.049）. The incidence rates of hemorrhagic cystitis were 57.1％ （4/7）and 8.6％ （3/35）, respectively （*P*＝0.005）, while the bacterial infection rates were 100％ （7/7）and 77.1％ （27/35）, respectively （*P*＝0.070）. The infection rates of EBV were 14.3％ （1/7）and 14.3％ （5/35）, respectively（*P*＝1.000）, while the incidence of fungal infection was 14.3％（1/7）and 25.7％（9/35）, respectively（*P*＝0.497）. The rates of post-transplant lymphoproliferative disease （PTLD）were 0％ and 5.7％, respectively（*P*＝0.387）.

**Conclusion:**

The findings of this study indicate that blood disease patients with G6PD deficiency can tolerate conventional allo-HSCT pretreatment regimens, and granulocytes and platelets can be implanted successfully. However, after transplantation, patients should exercise caution to avoid viral infection, complications of hemorrhagic cystitis, and secondary poor graft function.

葡萄糖-6-磷酸脱氢酶（G6PD）是红细胞戊糖磷酸途径的关键调控酶和活性胞质酶（cytosolic enzyme）[1]。G6PD基因突变可导致其活性不足，对氧化还原状态的调节作用减弱，G6PD缺乏可引起自由基介导的红细胞氧化损伤，导致过早溶血[2]。红细胞G6PD缺乏症是人类最普遍的X连锁酶病，男性相对常见，但女性携带者亦可有G6PD水平下降[3]。全球有3～4亿人患有不同程度的G6PD缺乏症，中国的发病率尚不清楚[4]。脐带血G6PD活性定量酶测定及荧光斑点试验显示，G6PD缺乏症在中国男、女性新生儿中的阳性率分别为4.4％、0.35％[5]。我国香港的一项研究显示，G6PD缺乏症在男性中的发病率为4.47％，女性为0.27％[6]–[7]。另一项研究发现，厦门G6PD缺乏症的患病率为1.39％[8]。与其他血液病合并存在时，G6PD缺乏会加重镰状细胞贫血患者的临床表现[9]–[10]，输血后G6PD缺乏的红细胞与间接胆红素轻度升高有关[11]。除了溶血，G6PD缺乏的不良影响已经在病毒感染、高胆红素血症/核黄疸、糖尿病、心血管疾病和神经退行性疾病中被报道[12]–[16]。但是G6PD缺乏症合并血液病患者异基因造血干细胞移植（allo-HSCT）的临床特征未见相关报道。在本研究中，我们对接受allo-HSCT的7例合并G6PD缺乏症血液病患者进行回顾性分析，探讨G6PD缺乏症患者与非G6PD缺乏症患者在allo-HSCT预处理毒性、移植后并发症和移植结局的差异。

## 病例与方法

1. 病例：本研究纳入2015年3月至2021年1月在北京大学人民医院接受allo-HSCT的合并G6PD缺乏血液病患者。使用“风险集抽样”（对于每个G6PD缺乏症病例，从同时期接受allo-HSCT的其他受试者中选择一组病例对照，以严格控制分析中时间的混杂效应），以1∶5的比例随机选择对照，匹配相同性别、移植年龄和移植适应证。共有7例G6PD缺乏症患者（研究组）和35例对照患者（对照组）纳入分析。

2. G6PD缺乏症的诊断：G6PD缺乏症的诊断流程包括G6PD高发地区新生儿足跟血测定干血滤纸片中的酶活性进行筛查、G6PD和6-磷酸葡萄糖酸脱氢酶（6GPD）活性比值法、基因检测等方法[17]–[18]。

3. 移植措施：患者的配型、供者选择、预处理方案、感染和移植物抗宿主病（GVHD）预防以及支持治疗均按我中心常规方案[19]–[25]进行。恶性血液病的预处理方案：①改良Bu/Cy方案（用于同胞全相合造血干细胞移植）：羟基脲80 mg/kg，−10 d；阿糖胞苷2 g/m^2^，−9 d；白消安3.2 mg·kg^−1^·d^−1^，−8 d～−6 d；环磷酰胺1.8 g·m^−2^·d^−1^，−5 d、−4 d；司莫司汀250 mg/m^2^，−3 d。②改良Bu/Cy+rATG方案：单倍体造血干细胞移植（haplo-HSCT）患者阿糖胞苷4 g·m^−2^·d^−1^，−10 d、−9 d；无关供者造血干细胞移植患者阿糖胞苷2 g·m^−2^·d^−1^，−10 d、−9 d；兔抗人胸腺细胞球蛋白（rATG）2.5 mg·kg^−1^·d^−1^，−5 d～-2 d；环磷酰胺剂量同上。③Bu/Cy/Flu+rATG方案（用于haplo-HSCT）：在改良Bu/Cy+rATG方案基础上，环磷酰胺总量减至2 g/m^2^（1 g·m^−2^·d^−1^，−5 d、−4 d）；氟达拉滨30 mg·m^−2^·d^−1^，−6 d～−2 d；白消安、rATG用法同上。④TBI/Cy/+ATG方案（haplo-HSCT）：全身放射治疗（TBI）770 cGy替代改良Bu/Cy+rATG方案的阿糖胞苷、白消安。重型再生障碍性贫血（SAA）患者的预处理方案：白消安3.2 mg·kg^−1^·d^−1^，−8 d、−7 d；环磷酰胺50 mg·kg^−1^·d^−1^，−5 d～−2 d；rATG 2.5 mg·kg^−1^·d^−1^，−5 d～2 d。

4. 定义：粒细胞植入：中性粒细胞计数（ANC）≥1×10^9^/L连续3 d；血小板植入：PLT>20×10^9^/L持续7 d且脱离血小板输注。移植物功能不良（PGF）：移植后28 d达到完全供者嵌合但骨髓增生低下且三系中至少2系达到以下标准：PLT≤20×10^9^/L、ANC≤0.5×10^9^/L、HGB≤70 g/L持续3 d以上伴红细胞输注依赖，同时排除活动性GVHD和原发病复发[26]。总生存（OS）时间定义为造血干细胞输注完成至末次随访或死亡的时间；无病生存（DFS）时间定义为从造血干细胞输注完成至血液病复发、末次随访或死亡的时间。

5. 随访：通过查阅住院和门诊病历及电话随访方式获得患者一般资料、疾病诊断和治疗、移植资料及出院后情况。随访截至2023年8月31日。

6. 统计学处理：分类变量组间比较采用Fisher精确检验，连续变量组间比较采用Student-*t*（正态分布）或Mann-Whitney *U*（非正态分布）检验。OS和DFS采用Kaplan-Meier曲线进行分析，采用Log-rank检验进行比较。双侧*P*<0.05认为差异有统计学意义。所有数据分析均使用SPSS 26.0软件进行。

## 结果

1. 患者基线特征：研究组及对照组的临床特征见表1。研究组7例G6PD缺乏患者中男6例，女1例，中位年龄37（2～45）岁。7例G6PD缺乏患者均来自高发地区，2例男患者出生后即确诊；4例男患者及1例女患者因阳性家族史于移植前行G6PD/6GPD检测并确诊，7例患者均无明显临床症状。所有供者G6PD/6GPD比值均在正常范围内。血液系统原发病包括急性髓系白血病（AML）3例、急性淋巴细胞白血病（ALL）2例、SAA 2例。

2. 移植特征：研究组男供男3例、女供男3例、男供女1例。5例急性白血病患者移植前均为完全缓解。haplo-HSCT 5例，无关供者、同胞全相合造血干细胞移植各1例。2例G6PD缺乏合并SAA患者以改良Bu/Cy+ATG方案进行预处理；5例G6PD缺乏合并急性白血病患者中4例以改良Bu/Cy+ATG方案、1例以改良Bu/Cy方案进行预处理。骨髓联合外周血干细胞移植4例，外周血干细胞移植3例。中位总有核细胞（MNC）输注量为10.23（7.83～12.92）×10^8^/kg，CD34^+^细胞输注量为3.6（2.0～10.0）×10^6^/kg。35例对照组患者的基线特征与G6PD缺乏患者相似，详见表1。

**表1:** 研究组（合并G6PD缺乏）与对照组（无G6PD缺乏）血液病患者的基线特征

特征	研究组（7例）	对照组（35例）	*P*值
性别［例（%）］			1.000
男	6（85.7）	30（85.7）	
女	1（14.3）	5（14.3）	
血液系统原发病［例（%）］			1.000
重型再生障碍性贫血	2（28.6）	10（28.6）	
急性髓系白血病	3（42.8）	15（42.8）	
急性淋巴细胞白血病	2（28.6）	10（28.6）	
白血病患者移植前疾病状态［例（%）］			0.384
完全缓解	5（100.0）	23（92.0）	
未完全缓解	0（0.0）	2（8.0）	
造血干细胞供者［例（%）］			0.831
单倍体	5（71.4）	21（60.0）	
同胞全相合	1（14.3）	8（22.9）	
无关	1（14.3）	6（17.1）	
造血干细胞来源［例（%）］			0.890
骨髓	4（57.0）	19（54.3）	
外周血	3（42.9）	16（45.7）	
预处理方案［例（%）］			0.706
改良Bu/Cy	1（14.3）	8（14.3）	
改良Bu/Cy+rATG	6（85.7）	25（80.0）	
Bu/Cy/Flu+rATG	0（0.0）	1（2.9）	
TBI/Cy/+rATG	0（0.0）	1（2.9）	
单个核细胞输注量（×10^8^/kg，*x±s*）	8.48±0.41	9.98±0.78	0.122
CD34^+^细胞输注量（×10^6^/kg，*x±s*）	3.52±0.44	5.19±1.12	0.089

注 G6PD：葡萄糖-6-磷酸脱氢酶；Bu：白消安；Cy：环磷酰胺；Flu：氟达拉滨；rATG：兔抗人胸腺细胞球蛋白；TBI：全身放射治疗

3. 粒细胞和血小板植入：研究组7例（100.0％）患者均在移植后28 d内获得粒细胞植入，对照组移植后28 d粒细胞植入率为94.3％。研究组、对照组粒细胞植入中位时间分别为13（11～17）d、12（10～23）d（*P*＝0.601）。血小板植入的中位时间分别为21（6～64）d、14（7～70）d（*P*＝0.113）。

4. 预处理相关毒性：所有G6PD缺乏患者在移植过程中均未发生溶血性贫血。1例女性患者在allo-HSCT后发生重症肺炎，因不能排除耶氏肺孢子菌感染，曾予口服复方磺胺甲唑2周，未出现溶血等不良反应。7例G6PD缺乏患者中有3例在预处理后出现肝损伤（42.9％），未发生肾和心脏损伤；对照组肝损伤发生率为31.4％，肾损伤发生率为8.6％，心脏损伤发生率为2.9％。以上预处理相关毒性均为1、2度，未发生3度及以上毒性。两组均未发生肺损伤和出血事件。预处理相关毒性在研究组和对照组之间差异无统计学意义。

5. 移植相关并发症：研究组4例、对照组13例患者发生急性GVHD，两组急性GVHD发生率相当。研究组Ⅰ度3例、Ⅱ度1例，未发生Ⅲ、Ⅳ度急性GVHD。对照组分别有6、6、1例患者发生Ⅰ、Ⅱ、Ⅲ度急性GVHD。急性GVHD发生率及分度在两组间差异无统计学意义。

研究组、对照组巨细胞病毒（CMV）感染发生率分别为71.4％（5/7）、31.4％（11/35）（*P*＝0.049），出血性膀胱炎发生率分别为57.1％（4/7）、8.6％（3/35）（*P*＝0.005），细菌感染发生率分别为100.0％（7/7）、77.1％（27/35）（*P*＝0.070），EB病毒（EBV）感染发生率分别为14.3％（1/7）、14.3％（5/35）（*P*＝1.000），真菌感染发生率分别为14.3％（1/7）、25.7％（9/35）（*P*＝0.497），PTLD发生率分别为0％、5.7％（2/35）（*P*＝0.387）。研究组未发生原发性PGF，继发性PGF发生率显著高于对照组［42.9％（3/7）对8.6％（3/35），*P*＝0.036］。

6. 生存与结局：随访截至2023年8月31日，研究组有1例患者死于严重感染合并移植相关血栓性微血管病。对照组有3例患者死亡，死亡原因包括严重感染（1例）和GVHD（2例）。

## 讨论

当G6PD缺乏症与血液病合并存在且需要接受allo-HSCT时，患者是否可耐受allo-HSCT预处理以及之后的合并症，既往缺乏相关报道。本研究结果显示，G6PD缺乏症患者移植过程中脏器损伤发生率无显著升高，患者可耐受常规预处理毒性，且粒细胞、血小板植入与非G6PD缺乏患者无显著差异，但移植后病毒感染、出血性膀胱炎、继发性PGF发生率显著升高，提示我们G6PD缺乏症患者接受allo-HSCT是安全可行的，但应加强对病毒感染、出血性膀胱炎和继发性PGF等合并症的防治。

G6PD通过葡萄糖-6磷酸氧化将NADP还原为NADPH的酶，然后NADPH参与谷胱甘肽还原酶将氧化的谷胱甘肽还原回其活性状态的过程，还原型谷胱甘肽可中和活性氧（reactive oxygen species, ROS），在抗氧化中起重要作用。ROS来源于人体正常新陈代谢、炎症或药物不良反应，当G6PD缺乏时，受到可导致ROS增加的因素如病毒感染、某些药物（磺胺类抗生素等）或食物（蚕豆等）刺激，会因ROS不能得到及时清除造成组织过氧化损伤，症状包括溶血性贫血、黄疸甚至肾衰竭[27]–[28]。在本研究中，所有合并G6PD缺乏的患者在移植过程中都避免了蚕豆、磺胺类抗生素等潜在溶血诱发因素的刺激，未发生溶血性贫血。在移植早期即使发生CMV/EBV感染或细菌、真菌感染，患者也没有出现G6PD缺乏的典型溶血症状，其中一例女性患者在发生重症肺炎后，因低氧血症，不能排除耶氏肺孢子菌感染，曾予口服复方磺胺甲唑，患者亦无溶血发作，这可能都得益于供者均为非G6PD缺乏患者，移植后G6PD活性逐渐恢复，但因本研究为回顾性资料，患者未进行G6PD监测，移植后亦未进行G6PD基因检测，需要未来更大宗病例或前瞻性研究证实该结论。

G6PD缺乏症患者除对特定食物、药物的过氧化刺激敏感外，因G6PD在免疫反应中也起到重要作用，G6PD患者感染风险显著增加[29]–[30]。中性粒细胞可通过直接吞噬和分泌中性粒细胞胞外诱捕网（NET）两种方式清除外来病原。研究发现，中性粒细胞可直接吞噬病毒，从而在多种病毒感染性疾病，如单纯疱疹病毒、CMV、流感病毒以及新冠病毒等发病中起到重要作用[31]–[35]。NET是活化中性粒细胞分泌的网状结构，负责捕获和杀死细胞外病原体，以防止微生物的传播，同时提醒免疫系统感染发生[36]–[38]。研究提示，G6PD缺乏时中性粒细胞吞噬病原以及分泌NET的功能都受到限制，从而削弱对病毒的清除能力[16],[30],[39]–[40]。此外，由于G6PD缺乏直接导致ROS增加，以及通过降低白细胞介素-1β和NLRP3炎症小体简介导致的ROS增加，都有可能有利于病毒复制，从而增加病毒清除的难度[41]。因此，G6PD缺乏患者病毒感染风险升高[42]。也有研究提示，G6PD缺乏症合并急性髓系白血病患者化疗后真菌感染的发生率显著增加[43]–[44]。本研究发现，G6PD缺乏症患者allo-HSCT后CMV感染和出血性膀胱发生率显著高于非G6PD缺乏患者。既往研究已发现，出血性膀胱炎是与BK病毒[45]、CMV[46]和腺病毒[47]感染等密切相关的allo-HSCT合并症。因此，G6PD缺乏症患者出血性膀胱炎发生风险升高也可能是其易发生病毒感染所致。但可能受限于病例数较少，本研究中EBV感染发生率在两组间差异无统计学意义。

继发性PGF在allo-HSCT中的发生率为3.4％～10％[48]–[50]。本研究中，对照组继发性PGF发生率为8.6％，与既往文献报道一致，但研究组继发PGF发生率高达42.9％。因为继发PGF的发生率与haplo-HSCT、急性GVHD以及CMV感染等密切相关[48]–[49],[51]，所以本研究中G6PD缺乏症患者移植后继发性PGF发生率升高可能是由于CMV感染、出血性膀胱炎等病毒感染发生率升高所致。

本研究结果显示，合并G6PD缺乏的血液病患者可耐受常规allo-HSCT预处理方案，粒细胞、血小板可顺利植入，但移植后需警惕病毒感染、出血性膀胱炎合并症及继发PGF。以上结论尚需更大规模研究以及前瞻性数据来证实。
